# Role of Microbial Infection-Induced Inflammation in the Development of Gastrointestinal Cancers

**DOI:** 10.3390/medicines8080045

**Published:** 2021-08-17

**Authors:** Keita Kouzu, Hironori Tsujimoto, Yoji Kishi, Hideki Ueno, Nariyoshi Shinomiya

**Affiliations:** 1 Department of Surgery, National Defense Medical College, Saitama 359-0042, Japan; dj27qd.t01312kk@gmail.com (K.K.); ykishi-3su@ndmc.ac.jp (Y.K.); ueno_surg1@ndmc.ac.jp (H.U.); 2 National Defense Medical College, Saitama 359-0042, Japan; shinomi@ndmc.ac.jp

**Keywords:** tumor progression, gastrointestinal cancer, microbial inflammation

## Abstract

There has been increasing evidence that a local inflammatory response stimulates tumor cells to acquire metastatic potential, and the concept of inflammatory oncotaxis has been spreading in recent years. However, the interaction between microbial inflammation and the development of gastrointestinal cancer is still unclear. This review summarizes the present knowledge on the role of microbial inflammation in the development of gastrointestinal cancers from the perspective of molecular biological findings. Chronic inflammation caused by bacterial infection is known to induce cancers as exemplified by Helicobacter pylori, which is associated with the development of gastric cancer via the activation of the TLR4 pathway by bacterial lipopolysaccharide followed by cancer growth through CagA-MET signaling. In addition, the development of inflammatory bowel diseases has been known to become a risk factor for colorectal cancers, where inflammation caused by certain bacterial infections plays a key role. It is also known that the cancer microenvironment is associated with cancer growth. Moreover, infectious complication after surgery for gastrointestinal cancers may promote tumor progression via the stimulation of pathogen-associated molecular patterns and various inflammatory mediators secreted by immunocytes. Further research on the link between microbial inflammation and cancer progression is needed to drive a paradigm shift in cancer treatment.

## 1. Introduction

Previous reports suggested that a persistent local inflammatory response stimulated tumor cells to acquire metastatic potential, and the concept of inflammatory oncotaxis has been spreading in recent years [1,2,3,4,5,6]. When local inflammation occurs, peripheral blood monocytes migrate to the site of inflammation, differentiate into macrophages, and produce various bioactive mediators which affect the growth and invasive capacity of tumor cells. Macrophages that exist in tumor tissues are called tumor-associated macrophages (TAMs), which have quite different activities from tissue-resident macrophages. TAMs produce many bioactive substances such as tissue growth factors, e.g., IL-6, TNFα, angiogenic factors, matrix metalloproteases (MMPs), and immunosuppressive factors, all of which are involved in cancer growth and metastasis. In addition, HGF is known to be produced after surgical stress and sepsis [7]. Molecular targeted therapeutics such as anti-EGFR, anti-VEGF, and PD-1/PD-L1 antibodies have been clinically applied in many carcinomas to inhibit angiogenesis and immune checkpoint functions, respectively [8,9,10,11]. The inhibition of MMPs may suppress cancer growth, angiogenesis, and extramedullary mobilization of bone marrow-derived cells [12]. Thus, it has been demonstrated that the host immune system is involved in tumor progression under the influence of inflammation, thereby attempts at clinical applications are underway. Conversely speaking, an increase in systemic inflammatory response is associated with poor long-term prognosis in many carcinomas [13,14]. The elevation of serum C-reactive protein levels is known to influence the production of inflammatory cytokines, and the state of high C-reactive proteins is associated with a higher mortality rate in people with gastrointestinal cancers [15]. In addition, the number of neutrophils in the blood circulation are also reflected in neutrophils infiltrating inside the tumor [16,17] Neutrophils in the tumor microenvironment is known to release various cytokines and chemokines [18]. Increased neutrophil counts as the result of myeloid cell recruitment and ectopic colony-stimulating factor production can result in inflammatory cell infiltration in the local tumor area followed by production of inflammatory cytokines. 

**Figure 1:**
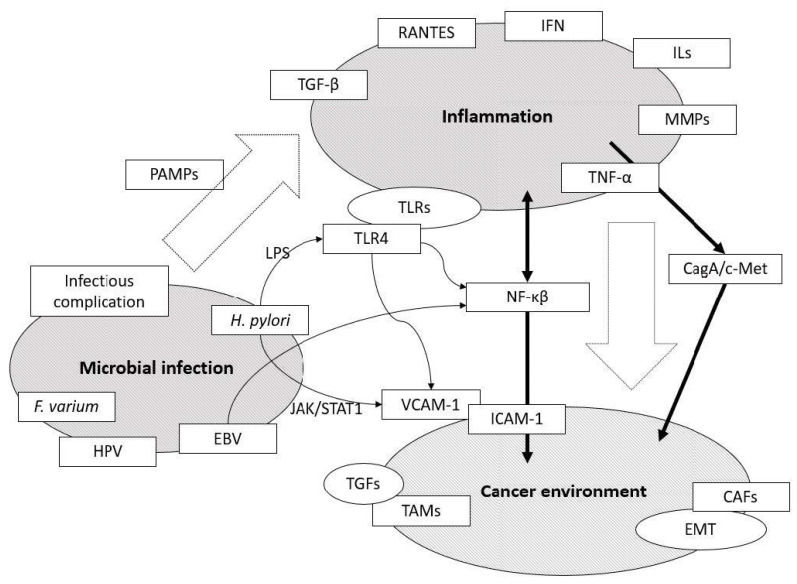
An overview of the links between microbial infection, chronic inflammation, and cancer environment. Several signaling pathways, such as TLR4 and CagA/c-Met, and mediators selected by microbial inflammation play an important role in the development of gastrointestinal cancers. EBV: Epstein-Barr virus; CAFs: Cancer associated fibroblasts; EMT: Epithelial mesenchymal transition; HGF: Hepatocyte growth factor; HPV: Human papilloma virus; IFN: Interferon; ILs: Interleukins; ICAM-1: Intercellular adhesion molecule-1; JAK/STAT1: Janus kinase/Signal transducer and activator of transcription 1; LPS: Lipopolysaccharide, MMPs: Matrix metalloproteinases; NF-κβ: nuclear factor-kappa β; PAMPs: Pathogen-associated molecular pattern molecules; RANTES: Regulated on activation, normal T cell expressed and secreted; TAMs: Tumor associated macrophages; TGFs: Transforming growth factor; TLR: Toll-like receptor; TNF-α: Tumor necrosis factor-α; VCAM-1: Vascular cell adhesion molecule-1.

When considering the mechanisms of cancer progression and metastasis under inflammatory conditions, it is essential to analyze the relationship between molecular changes associated with inflammatory microenvironment and how cancers are affected by them (Figure 1). Here in this review, we summarized current knowledge on the role of microbial inflammation in the progression and metastasis of gastrointestinal cancers with special reference to our latest findings.

## 2. Microbial Infection-Induced Gastrointestinal Cancers

Since Virchow reported the infiltration of leukocytes in cancerous tissues, numerous studies have been conducted on the relationship between cancer and inflammation [19]. It is said that infectious agents contribute to the etiology of about 15% of total gastrointestinal cancers and that chronic inflammation is a major component of infection-induced carcinogenesis [19]. In this section, factors directly linked to the development of gastrointestinal cancer were discussed.

### 2.1. Gastric Cancer

The microbial inflammatory response due to Helicobacter pylori infection is one of the best-understood models in the development of gastric cancer [20]. It is well known that patients who become infected with H. pylori and have typical histologic findings such as severe gastric atrophy are at high risk of gastric cancer [21,22]. H. pylori infection causes abnormal DNA methylation in gastric epithelial cells, which is also associated with high risk of carcinogenesis [23,24]. It is known that infection with H. pylori results in the activation of NF-κB pathway in myeloid cells that assists gastric epithelia in stimulating tumor progression and development [25]. H. pylori, microaerophilic Gram-negative bacteria possess lipopolysaccharides (LPS) in their cell wall and actively grow not only on gastric epithelial cells but also on gastric cancer cells. Toll-like receptor 4 (TLR4), a receptor for its ligand LPS, is more expressed in H. pylori-infected gastric epithelium than in non-infected epithelium [26]. Furthermore, it has been proven that H. pylori LPS promotes the proliferation of gastric cancer cells via the TLR4 pathway [26]. Similarly, it has been reported that the activation of TLR9 pathway and its expression via the cag pathogenicity island is directly related to gastric cancer risk [27]. Moreover, in gastric cancer associated with H. pylori infection, patients with MET-positive tumors were found to have a poorer prognosis than those with MET-negative disease [28]. MET is a receptor-type tyrosine kinase with multifaceted effects, including cell migration, survival, and proliferation, and has HGF as an endogenous ligand [29]. Therefore, HGF-MET signaling regulates cancer proliferation and invasion in the primary sites as well as subsequent growth in the metastatic lesions [30]. In MET-positive gastric cancer cell lines, H. pylori infection upregulates MET phosphorylation, and its downstream signals, such as p44/42 MAPK and Akt, are activated and confer gastric cancer cells upon proliferation and anti-apoptotic activities [28]. However, certain Lactobacillus species, including L. reuteri, L. johnsonii, and L. murinus, reportedly inhibit the growth of H. pylori in vitro and are symbiotic [31,32]. Experiments in mice have shown that gastric cancer is not caused by H. pylori alone but requires specific symbiotic bacteria, thereby playing a coordinated role in the process [33,34].

In addition, Epstein–Barr virus (EBV) has been reported to be involved in the development of 8–10% of gastric cancer [35,36]. EBV is a widespread pathogen to humans, which often causes several types of malignant lymphomas and nasopharyngeal carcinoma. Genes from EBV such as LMP-1 stimulate NF-kB signaling through the activation of TNF-receptor associated factors (TRAF) and constitutively activate cell proliferation signals and other latent membrane proteins playing important roles for activating Akt and ERK signals, which enhance cell survival. However, these signals are mainly involved in the progression of lymphomas. By contrast, EBV-nuclear antigens, especially EBNA1, suppress p53 dependent apoptosis and promote cell division of DNA damaged gastric epithelial cells, thereby resulting in the development of gastric cancer [36,37]. EBV genes have been known so far to activate oncogenic signaling pathways such as NF-κB, JNK, JAK/STAT, and PI3K/Akt [38]. In addition, a recent report has implied that EBV-associated gastric cancer cells with expressing PD-L1 suppressed T-cell proliferation and that the IFN-γ signaling pathway is involved in this expression of PD-L1 [39]. Therefore, EBV-associated gastric cancer may be a suitable target for immunoinhibitory checkpoint molecule [39].

### 2.2. Esophageal Cancer

Although the relationship between microbial inflammation and carcinogenesis of esophageal cancer remains unknown, there has been an increasing evidence of the involvement of human papillomavirus (HPV) infection in esophageal cancer [40]. HPV is a non-enveloped DNA virus which belongs to the papillomaviridae. HPV has over 100 genotypes; in particular, infections with HPV16 and HPV18 are well known to be the risk factor of squamous cell carcinomas such as cervical cancer, head and neck cancer, and anogenital tract cancer [41,42,43]. Research on the role of HPV in esophageal cancer mainly revealed the changes in cell cycle related genes and proteins such as p53 and p16 [44,45,46]. However, the molecular pathogenesis of esophageal cancer related to HPV infection still remains unclear in many aspects. One possibility is that E6 protein of HPV16 may promote the tumorigenesis of esophageal cancer via downregulating miR-125b expression, which results in the activation of Wnt/β-catenin signaling pathway [47]. Since studies about HPV in esophageal cancer have been increasing steadily, the elucidation of molecular biological mechanisms in this area is anticipated.

The development of esophageal cancer in the context of esophagitis is a good example of understanding the relationship between chronic and microbial inflammation. It is known that the normal esophageal flora is rich in Streptococcus viridans, a Gram-positive bacterium [48]. However, the fact that the microbiome of reflux esophagitis and Barrett’s metaplasia, a precursor condition to esophageal adenocarcinoma, has changed from being a predominantly Gram-positive to a mostly Gram-negative bacterial flora suggests that dysbiosis contributes to its pathogenesis [49]. LPS from Gram-negative bacteria can trigger the gene expression of proinflammatory cytokines via the activation of TLR4 and the downstream NF-kB pathways [50]. Dysbiosis in esophagitis might be associated with the cancer progression-related cytokine expression [51].

### 2.3. Colorectal Cancer

Colorectal cancer in inflammatory bowel diseases is considered one of the typical examples of inflammation-related cancers in which the epithelial microenvironment affects their growth capability.

First, the pattern of the bacterial flora on the colonic mucosa is suggested to be an important factor responsible for the persistence and aggravation of ulcerative colitis [52]. In inflammatory bowel diseases, the proportion of Proteobacteria containing several expression pathogenic bacteria tends to increase, whereas the phylum Firmicutes containing probiotics decreases in general [53]. For example, in patients with ulcerative colitis, Fusobacterium varium invading the host cells increases inflammatory cytokine, including IL-8 and TNF-α production [54]. It is well known that the risk of carcinogenesis in ulcerative colitis increases with the duration of exposure to inflammation [55]. Likewise, it is known that microbiome in patients with Crohn’s disease is different from that in healthy individuals. The relative amount of Bacteroidetes and Escherichia coli increased and Firmicutes decreased in Crohn’s disease [56].

Second, the pattern of bacterial flora on the colonic mucosa is also suggested to be an important factor responsible for developing colorectal cancer [57]. The role of Fusobacterium nucleatum, a Gram-negative anaerobic bacillus known as oral commensal bacterium, has received particular attention as a cancer-related member of the microbiota. A comprehensive DNA and RNA analysis showed that F. nucleatum over-representation is shown in colorectal cancer tissues relative to healthy tissues [58,59]. F. nucleatum detected in colorectal cancer has been shown to originate from the oral microbiome [60,61]. Moreover, F. nucleatum abundance is associated with a poorer prognosis and cancer recurrence of colorectal cancer due to resistance to chemotherapy [62,63]. It was reported that F. nucleatum activates TLR4 signaling and NF-kB and upregulates the expression of microRNA-21, resulting in their increased proliferation and invasive activity in colorectal cancer [64]. Moreover, specific TLRs, including TLR4, TLR5, TLR7, and TLR8, are known to be expressed in colon cancers [65]. Likewise, numerous solid cancer cell types also express TLRs, and both cancer TLRs and host-tissue TLRs activated by microbial infection augment cancer cell metastatic ability [66]. F. nucleatum also reportedly promotes colorectal cancer via the Wnt/β-catenin signaling [67,68]. Fecal F. nucleatum could be evaluated by quantitative PCR to improve the clinical utility of the fecal immunochemical test [69].

In addition, certain E. coli could be associated with colorectal cancer. E. coli with the polyketide synthase genotoxic island produces colibactin, causing cellular DNA damage and promoting carcinogenesis [70,71]. An attempt to detect colibactin-producing E. coli to diagnose colorectal cancer has been reported [72,73]. Colibactin-related colonic epithelial cell DNA damage reportedly results in tumor induction in patients with familial adenomatous polyposis [74]. Therefore, microbial inflammation also plays an important role in colorectal cancer carcinogenesis, and it could be a potential target for the treatment and prevention of colorectal cancer.

## 3. Interaction between Inflammation and Microenvironment

The microenvironment surrounding cancer stem cells is metabolically, functionally, and immunologically heterogeneous, which makes the treatment of diseases less effective by a simple standardized approach [75]. The stroma provides the microenvironment for cancer cells to promote differentiation into malignant forms called epithelial-mesenchymal transition (EMT) and tumor angiogenesis, which is essential for tumor nourishment. In particular, the mechanisms by which the mesenchymal stroma, composed of myofibroblasts such as cancer associated fibroblasts (CAFs), promotes cancer invasion and metastasis have been well studied [76,77]. Inflammatory mediators secreted by CAFs such as SDF-1, MCP-1/CCL2, IL-β, and RANTES/CCL5 induce immunosuppressive cells including myeloid-derived suppressor cells (MDSCs), regulatory T cells (Tregs), and tumor-associated macrophages (TAMs). Those cells play a key role to help cancer cells evade cytotoxic effects by NK cells and other immunocytes. Furthermore, CAFs produce a variety of humoral factors including HGF and PDGF-C, which activate tyrosine kinase signaling in neighboring cancer cells, thereby suppressing their sensitivity toward molecular targeted drugs against EGFR, BRAF, and VEGF [78,79,80]. In addition, healthy epithelial cells surrounding tumor cells might provide CAFs by inducing EMT in response to stimuli from the microenvironment [81,82].

The relationship between infectious inflammation and CAFs is not clear; however, it was reported that H. pylori infection activated VCAM-1 expression of CAFs in gastric carcinoma via the activation of JAK/STAT1 signaling pathway [83]. VCAM-1 is known to be induced by TLRs and proinflammatory cytokines and is related to cancer progression and metastasis of several cancer types [83]. Investigating the relationship between CAFs and inflammation caused by H. pylori may result in further research on gastric cancer and H. pylori.

## 4. Infectious Inflammation and Metastasis

Metastasis is the main character of malignant tumors in humans and is significantly associated with cancer death [84]. The multi-step process of cancer metastasis can be divided into the following two stages: The first stage is that cancer cells begin scattering from the primary tumor and invade the blood vessels, and the second stage includes adhesion to vascular endothelium and proliferation in the metastatic organ. The former requires the destabilization of epithelial cell–cell adhesion by reduced E-cadherin expression and MMP enzyme activity, thereby degrading collagen and other proteinous substances in the membrane and stroma [85,86]. The latter first requires the role of intercellular adhesion molecules, such as selectins and integrins, and then requires the aid of growth factors, including HGF and TGF-α, angiogenic factors such as VEGF, and local immunity [87]. Integrins are necessary for both tumor invasion and angiogenesis [88]. Certain integrins form signaling pathways with oncogenic receptor tyrosine kinases, including Met, EGFR, and HER2 [88,89]. VEGF enhances the expression and activation of several integrins, which induce angiogenesis [90]. Recently, the role of the tumor microenvironment is receiving attention on how inflammatory cells are composed and how it affects cancer growth by resembling chronic inflammation [91].

Although the most radical treatment for gastrointestinal cancer is surgical resection, the surgery itself is invasive, thereby causing severe inflammation and postoperative infectious complications such as anastomosis leakage, intra-abdominal abscess, and local infection at significant frequency. It has been suggested that these infectious complications not only affect the short-term postoperative outcome but also worsen the long-term prognosis. In fact, in many carcinomas, including esophageal, gastric, and colorectal cancers, patients who developed infectious complications after surgery showed significantly poorer long-term post-operative outcomes than those who did not [92,93,94]. By using animal models, we have previously reported that abdominal infection suppressed intrahepatic NK cell function and promoted liver metastases [95]. There has been accumulating knowledge that microbial pathogen-associated molecular patterns directly promote cancer progression [96]. Infectious complications stimulate the production of multiple mediators such as inflammatory cytokines and chemokines, and the complex involvement of those mediators is said to trigger a biological response in patients [97,98,99,100]. The overexpression of IL-10 and TGF-β associated with decreased IFN-γ and IL-12 production can reduce host tumor immunity and indirectly promote tumor growth [101,102]. Some cytokines/chemokines such as TNF-α, IL-18, and RANTES have been reported to be directly involved in tumor growth via the stimulation of their receptors expressed on the tumor cells [103]. Thus, inflammatory and immune responses in the patients are often associated with cancer growth, but infectious inflammation induced by postoperative complications is also associated with tumor progression [104]. Furthermore, we have found that the HGF/c-MET cascade was involved in liver metastasis formation by using a peritonitis animal model [105]. In the acute lung injury and pneumonia models, however, the involvement of HGF/c-MET was not obvious, and cell adhesion molecules such as ICAM-1 and E-selectin might rather be involved in lung metastasis [106]. These results demonstrated that the metastasis of tumor cells might be promoted through different mechanisms in different organs. Further elucidation of the mechanism of organ-specific tumor growth enhanced by infectious complications will result in new therapeutic strategies in each metastatic organ.

## 5. Future Perspective

The development of immune checkpoint inhibitors for cancer treatment has brought a paradigm shift in anti-cancer strategies. Therefore, precise and accurate understanding of host immunity and inflammatory complications associated with cancer is becoming more and more important. On the one hand, the suppression of the production of cytokines and chemokines in the cancer microenvironment, which plays a key role in cancer immunity, may also be an important therapeutic option in the future. On the other hand, the relationship between microbial inflammation and tumor microenvironment still remains unclear; thus, we still have many questions left to be answered, such as why infectious complications bring increased resistance to the treatment with molecular targeted drugs.

## Data Availability

No new data were created or analyzed in this study.
